# Editorial of Special Issue ‘Dissecting Neurological and Neuropsychiatric Diseases: Neurodegeneration and Neuroprotection’

**DOI:** 10.3390/ijms23136991

**Published:** 2022-06-23

**Authors:** Masaru Tanaka, László Vécsei

**Affiliations:** 1 ELKH-SZTE Neuroscience Research Group, Eötvös Loránd Research Network, University of Szeged (ELKH-SZTE), Semmelweis u. 6, H-6725 Szeged, Hungary; 2 Department of Neurology, Albert Szent-Györgyi Medical School, University of Szeged, Semmelweis u. 6, H-6725 Szeged, Hungary

This Special Issue has focused on dissecting the neuroprotective and neurodegenerative components of neurological and neuropsychiatric diseases, highlighting the latest advance in understanding the etiology, pathomechanism, biomarkers, imaging techniques, and novel therapeutic targets of neurodegenerative diseases (NDDs). The articles published in the Special Issue have featured Alzheimer’s disease (AD), Parkinsons’s disease (PD), multiple sclerosis (MS), dementia with Lewy bodies, and salicylate-induced tinnitus.

Broadly speaking, the disease onset and course may be roughly understood by imagining a tug-of-war between neuronal damage and recovery, which takes place as early as in prodromal phase. Then, who pulls the rope first? NDDs are multifactorial diseases in which genetic susceptibility, environmental factors, infections, nutrition, and/or lifestyle make a complex interaction to form an initial causative complex which later progresses to formation of secondary complex, eventually leading to the onset of diseases [1,2]. NDDs are characterized by impairments of both cognitive function and social interaction. Indeed, these alterations in social cognition and social functioning, are attributed to altered activity within cortical and subcortical brain structures [3], which store sensory, motor, and affective information, fundamental for self-awareness and decisional process [4], which is a crucial aspect in the symptomatology of various neurodegenerative disorders. Clinical features and changing functional patterns in NDDs include impairments in memory and emotional learning, poor planning, altered capacity to adapt behavior to the environment, impaired working memory, apathy, depression, disinhibition, and/or a dysexecutive cognitive profile, which correlate with a typical cognitive pattern due to frontal lobe dysfunction [4,5,6,7,8,9,10]. Thus, a certain interaction of etiological factors and a unique pathological progression together composite a team which triggers the initial pull of the rope. 

Featuring environmental factors as pathogenic culprits, Sini and colleagues explored the potential etiological link between microorganisms and NDD pathogenesis in an environmental scale. Blue-green algae cyanobacteria produce cyanotoxins which are released during cell lysis in an algal bloom into the surrounding water. Acute exposure to cyanotoxins cause gastrointestinal symptoms, allergic reactions, headache, and neurological symptoms including muscle weakness and dizziness. The cyanobacteria neurotoxin β-N-methylamino-l-alanine (BMAA) is considered to play a role in development of NDDs including AD, PD, and amyotrophic lateral sclerosis [11].

Diagnosis of a NDD is made through assembling a clinical picture interpreted by a doctor based on signs, symptoms, family history, and medical investigations including biomarkers, imaging tools, and medications [12]. The onset and disease course of NDDs may well be understood by envisioning a disease in analogy to a position vector: a position being the initial domain of symptoms; magnitude being severity in scales from molecular, tissue, neural correlate, to functional levels; and a direction being the domain of secondary symptoms. Neuroprotection, either through endogenous defense mechanisms or by exogenous supplements such as antioxidants may be able to intervene disease progression, slowing a free fall course with temporal remissions [13,14,15]. Following neural damage, direct repair mechanism neuroregeneration may help restore original or close to original brain functions in cellular and tissue levels [16]. Furthermore, overall neural activities can be maintained by the ability of the nervous system to recruit other components by reorganizing its structure, connections, and/or functions (that is neuroplasticity [17]). The capacity of neuroplasticity is bounded by resilience, which is the ability to be flexible and adaptive in response to harmful challenges [18]. The exacerbating disease course may well be exemplified by decreasing neural plasticity and weakening functional resilience. The endpoints of the plasticity, the resilience, and thus neural activities are neurodegeneration and eventually functional loss **(**Figure 1).

The initial neural damage can be repaired, and the function can be restored by the endogenous process of neuroprotection which refers to the salvage or recovery of the structure, function, neuronal cells, and/or network in the nervous system [19,20]. Microglial cells are responsible for inflammatory reaction in the nervous system. Czapski and Strosznajder discussed the roles of neuronal and microglial proteins including receptors, their involvement in the neural communication, and microglial-neuronal crosstalk in NDDs in search of neuroprotective and pharmacological targets [21]. The acute inflammatory response may proceed to neural recovery, but it may also lead to low-grade chronic inflammation and the state of immune tolerance. This is the allostatic state that maintains functional homeostasis at the cost of self-harm [22]. The healthy function in the network of excitatory glutamate and inhibitory γ-aminobutyric acid (GABA) neurotransmission is crucial to maintain neural homeostasis in the brain and the reciprocal excitatory-inhibitory balance has been observed to be compromised in neuroinflammation and AD [23]. Thus, the magnitude of organizational level is affected in AD through neuroinflammation.

Characterizing preclinical animal models simulating human diseases is an essential step for bench-to-bed translation research [24,25,26,27,28,29,30,31,32,33,34,35,36,37]. Mendes-Pinheiro and colleagues studied the behavioral domains of 6-hydroxydopamine (6-OHDA)-induced mouse model of PD. A progressive neurological disorder PD is widely considered to primarily affect movement of individuals. PD patients frequently experience psychological and behavioral symptoms named non-motor symptoms, which include sensory complaints, mental disorders, sleep disturbances, autonomic dysfunction, peripersonal space coding difficulties, motor disfunction and psychobehavioral symptoms such as apathy, agitation, hypersexuality, pathological gambling, psychoses, hallucinations, depression, and anxiety [2]. These symptoms can be present in the early stages of the disease, sometimes even before the appearance of classical motor symptoms, likely in relation to dopamine depletion in basal ganglia, suggesting how modulation of autonomic nervous system responses is fundamental for behavioral regulation. Evidence in healthy participants may suggest that these proprioceptive and motor mechanisms might be impaired in PD patients [38,39]. The authors investigated not only motor and coordination domain, but also the domains of positive and negative valences together with glial cell response. The mice showed despair-like behavior, decreased self-care, and less motivational behavior with proliferative and reactive microglia [40]. Thus, the authors successfully characterize a pharmacological animal model of PD, which also manifests the directional component of neural damage vector. 

Biomarkers are measurable indicators to help evaluate risk, diagnosis, disease course, and therapeutic outcomes for a disease. MS is a chronic immunological neurodegenerative disease of which biomarkers certainly may be able to help identify various stages of MS and build personalized treatment plan. Biernacki and colleagues reviewed promising new biomarkers of blood and cerebrospinal fluid samples for MS, emphasizing need to develop biomarkers from blood samples and to establish biomarkers to complement current diagnostic strategies [41]. Furthermore, discovery of prodromal biomarkers is an urgent need not only for MS, but other NDDs in order to prevent the onset of diseases. Development of new diagnostic imaging techniques is under extensive research for NDDs. The accumulation of the tau protein is closely linked to neurodegeneration and thus cognitive impairment. The tau protein may serve as diagnostic and prognostic biomarkers. However, its precision calibration in vivo remained a great challenge. Ricci and colleagues reviewed recent advances in in vivo imaging by positron emission tomography (PET) using tau tracer 2-Deoxy-2-[18F]fluoroglucose (18F-FDG), describing the development of tau PET tracers and the distribution of tau tracers and pattern in the brain [42]. Accordingly, blood biomarkers and in vivo imaging techniques potentially serve as barometers of sustainability in the brain including neural plasticity and functional resilience.

Neuropeptides and neurohormones play an important role in cognitive, emotional, social, and arousal functions. Thus, neuropeptide fragments, receptor antagonists, and analogues are under extensive study in search of their beneficial use for neurological and psychiatric diseases [43,44,45,46]. Kisspeptin is a neuropeptide that plays a crucial role in the function of the hypothalamic–pituitary–gonadal axis. Simon and colleagues reported that A kisspeptin fragment Kisspeptin 10 (K-10) was observed to mitigate amyloid-β toxicity by direct biding. Simon and colleagues showed that low concentrations of KP-10 suppressed wild-type and E46K mutant α-syn-mediated toxicity in vitro and that KP-10 favorably binds to the active sites of wild-type and E46K 32 mutant α-syn in silico, concluding that KP-10 may be a potential therapeutic agent targeting the active sites of α-syn [47].

Drug repurposing is the fastest and the most economical drug developing strategy screening already approved drugs for other medical conditions in search of new indications [48]. Valproic acid (VA) is primarily indicated for epilepsy, bipolar disorder, and prophylaxis of migraine and seizures [49]. Song and colleagues investigated the neuroprotective effects of VA on an ototoxic drug salicylate-induced tinnitus model in vitro and in vivo, showing that salicylate-induced excitotoxicity and production of reactive oxygen species were attenuated by VA [50]. The study presented exogenous use of neuroprotective agent for temporary tinnitus and healing loss.

Accordingly, this Special Issue has successfully presented research articles covering a broad aspect of NDDs from environmental etiological factors, cellular and functional pathomechanism, preclinical models, clinical biomarkers, imaging technique, and endogenous neuroprotective peptides to exogenous neuroprotective medicine. Identifying combinations of multifactorial factors which increase susceptibility to an NDD including the initial causative and the subsequent causative complexes and detecting such risk factors in a prodromal phase would be of particular interest for prophylactic measures. Revealing the pathomechanism of NDD exacerbation, calibrating the levels of neuroplasticity, and measuring the strength of functional resilience would help plan personalized treatment to induce and secure remission. Monitoring the status of tryptophan-kynurenine metabolism which has close ties with neuroinflammation may shed some light on these approaches to neuropsychiatric symptoms [51,52,53,54,55,56,57,58]. Exploring endogenous and exogenous neuroprotective approaches would be able to complement current disease-modifying strategies and thus may be able to delay development of full-blown neurodegeneration and functional loss in NDDs.

## Figures and Tables

**Figure 1:**
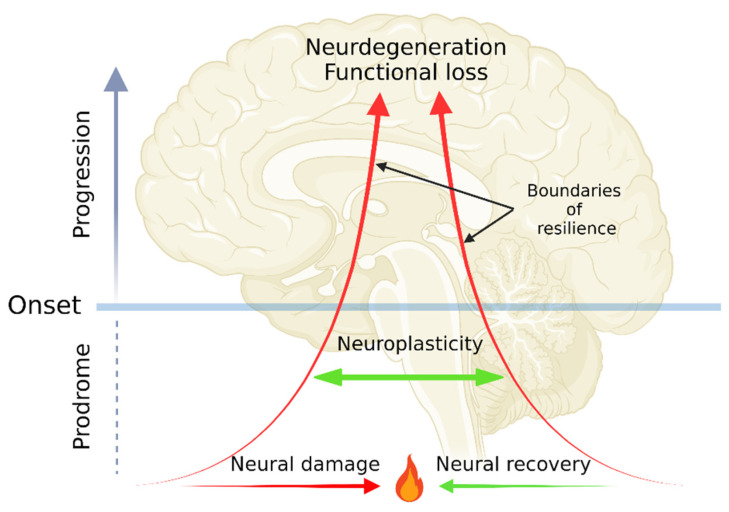
The dynamics of neurodegenerative diseases (NDDs) from prodromal phase, onset, neurodegeneration, to functional loss. The pathogenesis of NDDs starts from a tug-of-war between neural damage and neural recovery in prodromal phase. The normal neural functions can be compensated by neuroplasticity, which is bounded by functional resilience. Decreasing neuroplasticity and resilience lead to neurodegeneration and functional loss. The figure was created with BioRender.com.

## Abbreviations

AD	Alzheimer’s disease
BMAA	β-N-methylamino-l-alanine
18F-FDG	2-Deoxy-2-[18F]fluoroglucose
GABA	γ-aminobutyric acid
K-10	Kisspeptin 10
MS	multiple sclerosis
NDD	neurodegenerative disease
6-OHDA	6-hydroxydopamine
PD	Parkinson’s disease
PET	positron emission tomography
VA	Valproic acid
